# MMP9-Associated Tumor Stem Cells, CCL1-Silenced Dendritic Cells, and Cytokine-Induced Killer Cells Have a Remarkable Therapeutic Efficacy for Acute Myeloid Leukemia by Activating T Cells

**DOI:** 10.1155/2023/2490943

**Published:** 2023-05-09

**Authors:** Min Dong, Guozhen Zhang, Jie Meng, Biou Liu, Duanfeng Jiang, Feng Liu

**Affiliations:** ^1^Department of Hematology, The Second Affiliated Hospital of Hainan Medical University, Haikou 570000, China; ^2^Department of Hematology, The Fourth Affiliated Hospital of Harbin Medical University, Harbin 150001, China; ^3^Department of Hematology, The Affiliated Hospital of Guilin Medical University, Guilin 541001, China

## Abstract

**Purpose:**

Dendritic cells (DC) are specialized antigen-presenting cells, and cytokine-induced killer (CIK) cells have a specific killing activity to a variety of tumors. However, the underlining mechanism and function of DC-CIK cells in acute myeloid leukemia (AML) remain largely elusive.

**Methods:**

Gene expression profiles of leukemia patients were obtained from TCGA, DC cell components were evaluated using the quanTIseq method, and cancer stem cell scores were estimated using machine learning methods. The transcriptomes were obtained in DC-CIK cells from normal and AML patients by high-throughput sequencing. Large differentially expressed mRNAs were verified by RT-qPCR assay, and MMP9 and CCL1 were selected for subsequent studies *in vivo* and *in vitro* experiments.

**Results:**

Significant positive correlations were found with DC versus cancer stem cells (*p* = 0.008) and the expression of MMP9 versus cancer stem cells (*p* = 0.018). MMP9 and CCL1 were found to be highly expressed in DC-CIK cells from AML patients. DC-CIK cells with MMP9 and CCL1 knockout alone had little effect on leukemia cells, while knockdown of MMP9 and CCL1 in DC-CIK cells increased cytotoxicity, suppressed proliferation, and induced apoptosis of leukemia cells. In addition, we proved that MMP9- and CCL1-silenced DC-CIK cells significantly elevated the CD^3*+*^CD^4*+*^ and CD^3*+*^CD^8+^ cells and lowered the CD4^+^PD-1^+^ and CD8^+^PD-1^+^ T cells. Meanwhile, blockage of MMP9 and CCL1 in DC-CIK cells dramatically increased IL-2 and IFN-*γ*, increased CD107aþ (LAMP-1) and granzyme B (GZMB), and downregulated PD-1, CTLA4, TIM3, and LAG3 T cells from AML patients and AML model mice. Furthermore, activated T cells in DC-CIK cells knocking down MMP9 and CCL1 also prevented proliferation and accelerated apoptosis of AML cells.

**Conclusion:**

Our findings demonstrated that blockage of MMP9 and CCL1 in DC-CIK cells could markedly enhance the therapeutic efficiency in AML via activating T cells.

## 1. Introduction

Acute leukemia (AL) is a malignant clonal blood system disease derived from hematopoietic stem/progenitor cells [1]. Due to the rapid progress and short natural course of the disease, AL is a highly malignant blood tumor that endangers the life of the patient [2]. AL is divided into acute myeloid leukemia (AML) and acute lymphoblastic leukemia (ALL) [3]. Among them, AML accounts for more than half of AL cases, and among the AML subtypes, M2 and M5 had the highest incidence [4]. Currently, the etiology and pathogenesis of AML are still unclear, and the known related factors include infection, ionizing radiation, genetic factors, chemical substances, and immune abnormalities [5, 6]. In recent years, due to the application of high-dose chemotherapy, the 5-year overall survival (OS) rate of patients younger than 60 years is about 40%, and the OS rate of patients older than 60 years is less than 10% because of the recurrence [4]. Although great progress has been made in AML through the applications of new drugs, such as CD33 monoclonal antibody, DNA methyltransferase inhibitors, FLT-3 transplantation factors, and histone deacetylase inhibitors, the curative effect is still not ideal [7]. Currently, the best treatment strategy for AML patients is to combine new cytotoxic drugs, new molecular-targeted drugs, and immunotherapy with standard therapy [8, 9]. Among them, immunotherapy has significant effects in activating the immune system and inducing the immune response against leukemia [10]. Therefore, it is essential to study the pathogenesis of AML based on immunotherapy, so as to find new treatment strategies and means.

Cytokine-induced killer (CIK) cells are a class of mixed cell population with significant antitumor effects [11]. The antitumor mechanisms of CIK cells include the following: CIK cell directly kills tumor cells through the combination of CD3 and T-cell antigen receptors to induce the release of toxic particles in CIK cells. CIK cells kill tumor cells by secreting various cytokines including IL-2, IL-4, IL-6, IL-10, INF-*γ*, GM-CSF, and TNF-*α*. CIK cells induced apoptosis of tumor cells [12–14]. Dendritic cells (DC) are the most powerful antigen-presenting cells with the highest immune function, which can activate the initial T cells to trigger a strong anti-tumor-specific immune response in the body [15, 16]. It has been confirmed that DC can increase the activity of CIK cells, because DC can prominently accelerate the secretion of IFN-*γ* in CIK cells; increase CD3^+^CD4^+^, CD3^+^/CD8^+^, and CD3^+^CD56^+^ cells; and reduce CD4^+^CD25^+^ Treg cells through antigen presentation [17, 18]. Studies have also testified that DC can improve the proliferation of CIK cells. DC can further induce CIK cells to release more cytokines, such as IL-2, IL-12, and IFN-*γ*. The coculture of DC and CIK cells can also markedly enhance the antitumor activity and cytotoxicity of CIK cells. Dendritic cells and cytokine-induced killer (DC-CIK) cells also can notably reduce the secretion of immunosuppressive T cells (Treg cells) and IL-10 [19, 20]. At present, DC-CIK cells, as an emerging cellular immunotherapy, have significant advantages in the treatment of a variety of cancers, which can greatly improve the survival rate of patients [21–23]. However, the specific functions and mechanisms of DC-CIK cells in AML are not fully understood.

Currently, the RNA sequencing has become a comprehensive and accurate method for analyzing gene expression patterns [24]. Compared with other techniques, RNA sequencing can analyze the complexity of eukaryotic transcriptomes, with smaller deviations, lower false-positive rates, and higher reproducibility [25]. Therefore, in our study, RNA sequencing was selected to explore the complexity of transcriptomes in DC-CIK cells from normal and AML patients, which might lay the foundation for screens of candidate genes related to AML in DC-CIK cells. Moreover, we further investigated the impacts of MMP9- and CCL1-silenced DC-CIK cells on the proliferation and apoptosis of AML cells and the cocultured AML cells with the activated T cells. Meanwhile, we further verified the influences of MMP9- and CCL1-silenced DC-CIK cells on the T-lymphocyte function and the activation and depletion of T cells in the T cells cocultured with AML cells and AML model mice.

## 2. Materials and Methods

### 2.1. Clinical Samples

AML patients admitted to The Second Affiliated Hospital of Hainan Medical University and patients without hematological malignancies were included in this study. All AML patients were confirmed by morphological, immunological, cytogenetic, and molecular biology (MICM) examinations. Diagnosis and classification of patients were based on the revised French-American-British (FAB) classification and the 2008 World Health Organization (WHO) criteria [26, 27].  Table 1 summarizes the detailed clinicopathological characteristics of the patients. Before blood collection, we have obtained the informed consent from the patients, and our study was approved by the hospital ethics committee.

### 2.2. Cell Culture

Mononuclear cells (PBMCs) were collected from the peripheral blood of AML patients by density gradient centrifugation. Cells were cultured in RPMI-1640 medium (Gibco) containing 10% FBS and penicillin/streptomycin for 4 h. Subsequently, a medium containing 500 U/mL IL-4, 500 U/mL GM-CSF, and 500 U/mL TNF-*α* was added to the adherent cells to induce monocytes to differentiate into DCs. 1000 U/mL IFN-*γ*, 300 U/mL IL-2, and 50 ng/mL anti-CD3 monoclonal antibody were added to the suspension cells to induce CIK cell formation. DCs cultured for 7 days were harvested, the cell concentration was adjusted to 1 × 10^6^ cells/mL, and DC-CIK cells were cocultured at a ratio of 1 : 10 to generate DC-CIK cells.

DC-CIK cells continued to be amplified and activated *in vitro*. K562 (AML cell line), Jurkat (all cell line), and HL-60 (AML cell line) are leukemia cell lines obtained from ATCC. All three types of cells were cultured in RPMI-1640 at 37°C, 5% CO_2_, and 10% FBS (Thermo Fisher Scientific). PBMCs from clinical samples were washed with a serum-free medium. CD4^+^ and CD8^+^ T cells were separately isolated from PBMCs by immunomagnetic depletion using human CD4^+^ and CD8^+^ T-cell isolation kits according to the manufacturer's instructions (Miltenyi Biotec). T-cell purity and viability determined by flow cytometry analysis exceeded 95% and 98%, respectively. T-cell isolation in DC-CIK cells was the same as in PBMCs. AML cells were cocultured with DC-CIK cells at a ratio of 1 : 10. AML cells were cocultured with T cells at a ratio of 1 : 10. The viability of AML cells was detected after 3 days of treatment.

### 2.3. RNA Sequencing

DC-CIK cells from normal and AML patients were collected, and total RNA was extracted with TRIzol reagent (Invitrogen) based on the standard procedures provided by the manufacturer. After purification, total RNA was applied for library construction, and the concentrations and sizes of libraries were confirmed. The library was quantitatively detected using Illumina HiSeq 4000 System. The differential genes between the samples were analyzed using the edgeR software. The fold changes were counted using fragments per kilobase million (FPKM).

### 2.4. Cancer Stem Cell Analysis

The unified and standardized pan-cancer dataset was downloaded from the TCGA database. The deconvo_quantiseq method [28] of the R software package IOBR (version 0.99.9) [29] was used to evaluate the expression profile to predict the components of dendritic cells. The EREG.EXPss cancer stemness score calculated by mRNA characteristics for each tumor obtained from previous studies [30] was analyzed for Pearson's correlation between DC and cancer stem cell scores for each patient.

### 2.5. GO Analysis

Based on previous researches [31, 32], GO enrichment analysis was conducted through the DAVID (version 6.8, https://david.ncifcrf.gov/).

### 2.6. KEGG Analysis

As reported in the literature [33], KEGG analysis created by Kanehisa Laboratories was conducted through the KOBAS 2.0 software (http://kobas.cbi.pku.edu.cn/).

### 2.7. Cell Transfection

MMP9 and CCL1 shRNAs and negative control (NC) shRNAs were purchased from GenePharma (Shanghai, China). The sequences of MMP9 shRNAs were 5′-GCATAAGGACGACGTGAATGG-3′ and 3′-CCATTCACGTCGTCCTTATGC-5′; the sequences of CCL1 shRNAs were 5′-GCTCGCGAGCTATAGAAGAAT-3′ and 3′-ATTCTTCTATAGCTCGCGAGC-5′. DC-CIK cells (1 × 10^5^ cells/well) were inoculated in 6-well plates. After incubation for 12 h, DC-CIK cells were transfected with NC shRNAs or MMP9 and CCL1 shRNAs by the application of lipofectamine 3000 reagent (Invitrogen) based on the instructions, respectively.

### 2.8. Cell Proliferation

For the cell viability assay, the transfected DC-CIK cells or T cells and cocultured leukemia cells were plated into a 96-well plate and incubated for 24, 48, and 72 h. Then, cells were incubated in 10% CCK-8 working solution (5 mg/mL, Beyotime Institute of Biotechnology, Shanghai, China) in the dark for 1 h. The absorbance at 450 nm was recorded using a microplate reader (BioTek Instruments, Winooski, VT, USA). The evaluation of DC-CIK cells or T cells and cocultured leukemia cell proliferation by EdU staining was finished using the BeyoClick EdU-647 Cell Proliferation kit (Beyotime Biotechnology, Beijing, China) following the manufacturer's protocol.

### 2.9. Flow Cytometry

DC-CIK cells in each group were digested and suspended, and the cell density was adjusted to 1 × 10^6^ cells/mL. The 1 mL cells was washed with PBS through centrifugation (1000 × *g* at 4°C for 10 min). For apoptosis, the cell precipitate was then resuspended with 200 *μ*L binding buffer, and cells were disposed of 10 *μ*L Annexin V-FITC and 5 *μ*L PI for 15 min away from the light. For double dye, after cocultivation with T cells, the treated BMDCs (1 × 10^6^ cells/mL) were added with 5 *μ*g FITC-labeled CD4 and 5 *μ*g FITC-labeled CD8 or 5 *μ*g FITC-labeled PD-1 and 5 *μ*g FITC-labeled CD8 for 30 min. The results were examined through flow cytometry, and cell percentage was counted with the CellQuest software (BD Biosciences).

### 2.10. ELISA Assay

In accordance with the instruction provided by the supplier, the levels of IL-2, IFN-*γ*, GZMB, LDH, and LAMP-1 were confirmed using a Mouse IL-2 ELISA Kit (Beyotime, Cat. No. PI575), Mouse IFN-*γ* ELISA Kit (Beyotime, Cat. No. PI508), Mouse GZMB (Granzyme B) ELISA Kit (Elabscience, Cat. No. E-EL-M0594c96T), Mouse LDH ELISA Kit (Beyotime, Cat. No. PI596), and Mouse LAMP-1 ELISA Kit (Cat. No. EM1182).

### 2.11. Quantitative Reverse Transcriptase PCR (qRT-PCR) Assay

Total RNA was acquired with TRIzol reagent (Takara, Cat. No. 9109). After the extracted RNA was identified, cDNA was synthesized by applying Bestar™ qPCR RT kit (DBI, Cat. No. 2220). Then, the levels of genes were confirmed through amplification with Bestar™ qPCR MasterMix (DBI, Cat. No. 2043). The data were processed by 2^−△△CT^ method, and the primers were displayed in Table 2.

### 2.12. Western Blot Assay

The total proteins were obtained with RIPA lysate buffer (Beyotime) including a protease inhibitor, and the concentration was analyzed through the BCA method. 40 *μ*g proteins was denatured by heating at 100°C for 3 min and then was separated on 10% SDS-PAGE (Solarbio) through electrophoresis. After transfer to the PVDF membrane (Millipore), the proteins were blocked with 5% skimmed milk and addressed with primary antibodies overnight at 4°C and HRP-secondary antibody (Cell Signaling Technology) for 2 h. After treatment with an ECL kit (Thermo Scientific), the results were confirmed through ChemiDoc-X (Bio-Rad).

### 2.13. Animal

Six to eight weeks of male BALB/c mice were from the experimental animal center of Southern Medical University. The feed conditions included temperature (22-24°C), humidity (50%-60%), 12 h artificial light, standard feed, and sterile water. This experiment was conducted in line with the Institutional Animal Protection and Ethics Committee of the Second Affiliated Hospital of Hainan Medical College. HL-60 cells (5 × 10^4^ cells/mL) were injected into mice through the tail vein to establish the AML mouse model. In this study, the proportion of leukemia cells in mouse blood smear and bone marrow smear was observed by Wright's staining method to determine the successful establishment of the AML model [34, 35]. After 24 h, the modified DC-CIK cell (5 × 10^4^ cells/mL) was also injected into each group through the tail vein. The mice were randomly assigned into 7 groups: control, AML, AML+DC-CIK, AML+DC-CIK_sh-NC_, AML+DC-CIK_sh-MMP9_, AML+DC-CIK_sh-CCL1_, and AML+DC-CIK_sh-MMP9+sh-CCL1_ groups, 8 mice in each group.

### 2.14. Flow Cytometry of Antibody Markers in Whole Blood

Mice were anesthetized with sodium pentobarbital at a dose of 45 mg/kg. After injection, 100 *μ*L of peripheral blood was harvested from the inner canthus vein. The peripheral blood was added to the flowing antibodies CD3, CD8, CD4, PD-1, and TIM3 and thoroughly mixed. After incubation for 30 min at 4°C in the dark, the cell solution in each group was added with 3 mL erythrocyte lysate for 7 min. After centrifugation and suspension, the cells were detected by flow cytometry.

### 2.15. H&E Staining

All mice were euthanized by injection of sodium pentobarbital at a dose of 135 mg/kg. The spleen of the mouse was dissected. The spleen tissues were fixed with 4% paraformaldehyde, dehydrated with gradient alcohol, and embedded in paraffin. Then, the tissues were cut into 4 *μ*m slices. After dewaxing, the spleen tissues were processed with xylene I, xylene II, gradient ethanol, and distilled water. Next, the slices were addressed with the Harris hematoxylin, 1% hydrochloric acid alcohol, 0.6% ammonia, and eosin. After dehydration and transparency, the tissue morphology of each group was observed using a light microscope.

### 2.16. Immunochemistry Assay

Similarly, after dewaxing and hydration, the slices are processed with 3% hydrogen peroxide and EDTA (pH = 8), respectively. Subsequently, the slices were dealt with primary antibodies (anti-PD-1, anti-TIM3, anti-LAG3, and anti-CTLA4) at 37°C for 1 h. After incubation with a secondary antibody for 30 min, the slices were treated with 3,3′-diaminobenzidine (DAB), hematoxylin, and 0.1% hydrochloric acid. We finally observed and photographed the results with a light microscope after dehydration.

### 2.17. Statistical Analysis

All data in the current study were presented as mean ± SD. 20.0 SPSS software (PSS, Inc., Chicago, USA) was applied to analyze the experimental data. The results were also counted with *t*-test (two groups of comparison) or one-way analysis of variance (more than two groups of comparison). *P* < 0.05 represented statistically significant.

## 3. Results

### 3.1. RNA Sequencing Analysis in DC-CIK Cells from Normal and AML Patients

**Figure 1:**
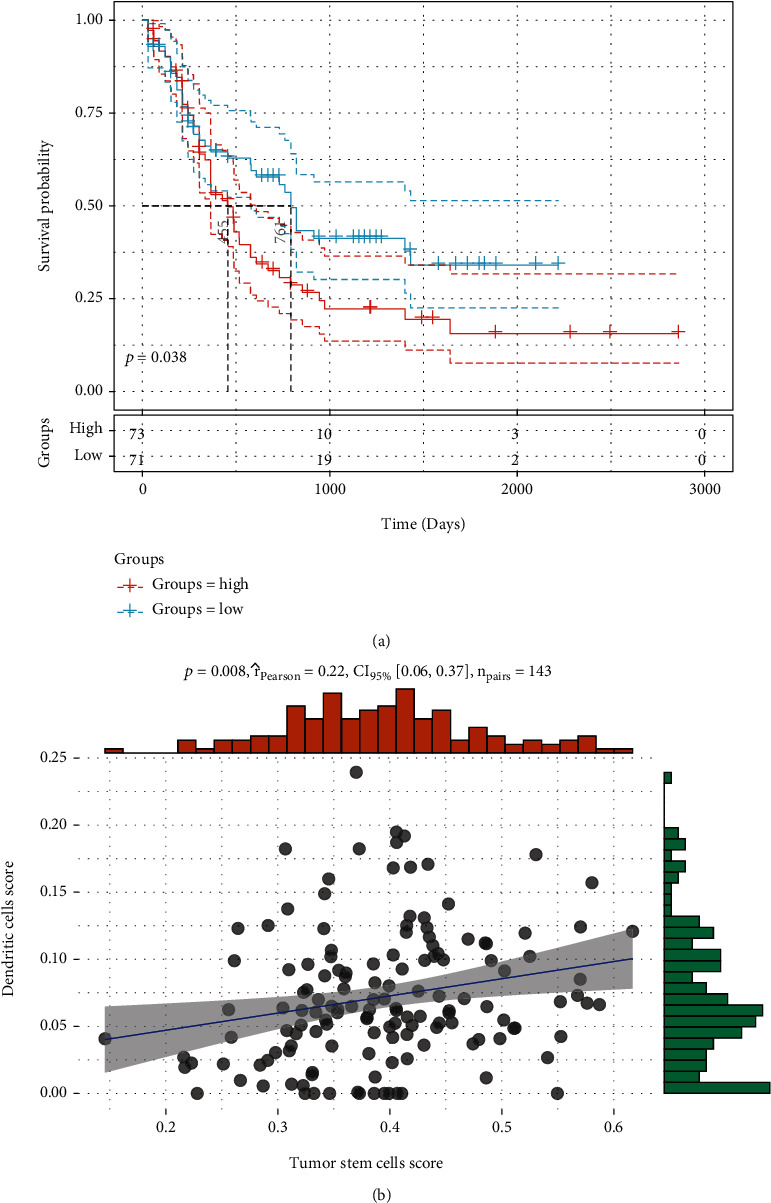
Cancer stem cell analysis. (a) Prognostic difference between patients with high and low DC in tumor microenvironments. (b) Correlation between DC components and tumor stem cell components in tumor microenvironment.

**Figure 2:**
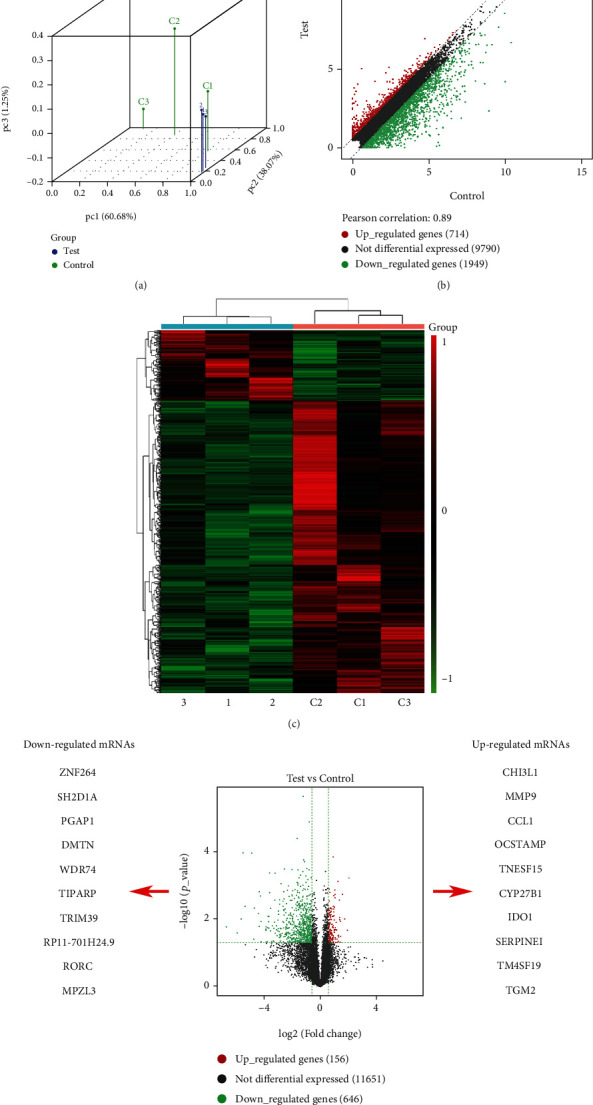
RNA sequencing analysis in DC-CIK cells from normal and AML patients. (a) After RNA sequencing, the principal component analysis was utilized to display the classification of expression profile data between the DC-CIK cells from normal and AML patients. The colors represent groups, and the *y*-axis represents a principal component. (b) The expressions of mRNAs were exhibited using a scatter diagram. (c) Heat map was also used to display the differentially expressed mRNAs in the DC-CIK cells from normal and AML patients. (d) Volcano plot was also adopted to represent the upregulated (red dots), not differentially expressed (black dots), and downregulated (green dots) mRNAs.

To analyze the relationship between DC cells and tumors in AML, we first evaluated the DC components of each patient in the TGCA-LAML cohort, and we observed that patients with a higher proportion of DC had a significantly worse prognosis (Figure 1(a)). Cancer stem cells are known to play a critical role in the survival, proliferation, metastasis, and recurrence of leukemia cells. We evaluated the relationship between cancer stem cells and DCs and observed that DCs showed a significant positive correlation with cancer stem cells (Figure 1(b)). These results suggest that DC is associated with poor prognosis in AML patients. To identify the anomalously expressed mRNA DC-CIK cells between normal and AML patients, PBMCs were isolated and DC-CIK cells were prepared. Subsequently, the differentially expressed mRNAs in DC-CIK cells were examined through RNA sequencing. The classification of the expression profile data was presented through a boxplot of APC to intuitively compare the mRNAs in different samples (Figure 2(a)). Meanwhile, the population distribution of differentially expressed data was displayed through the scatter plot, and we discovered that there were 714 upregulated genes (red dots), 1949 downregulated genes (green dots), and 9790 not differentially expressed genes (black dots) (Figure 2(b)). Similarly, we also display the differentially expressed mRNAs using the Heat map in the DC-CIK cells from normal and AML patients (Figure 2(c)). Next, volcano plot was applied to show the 156 upregulated genes (red dots), 11651 not differentially expressed genes (black dots), and 646 downregulated genes (green dots) (Figure 2(d)). As shown in Figure 2(d), we screened 10 genes with the most upregulated and downregulated multiples, respectively, according to the sequencing information (Figure 2(d)). Besides, the related functions of the differentially expressed genes were also commented via the usage of GO analysis in the DC-CIK cells. We found that the upregulated expressed genes were mainly enriched in biological process (BP) terms (cellular process, regulation of cellular process, response to stimulus, etc.), cellular component (CC) terms (cell, intracellular, cytoplasm, etc.), and molecular function (MF) terms (amide binding, peptide binding, protein binding, etc.); the downregulated expressed genes were mainly enriched in BP terms (metabolic process, cellular metabolic process, primary metabolic process, etc.), CC terms (intracellular, membrane-bounded organelle, intracellular organelle, etc.), and MF terms (protein binding, peptide binding, amide binding, etc.) (Supplementary figure 1A). The data of KEGG analysis also demonstrated that the enrichment pathways of the upregulated genes mainly included carbon metabolism (23 genes), glycolysis/gluconeogenesis (17 genes), and biosynthesis of amino acids (15 genes); the enrichment pathways of the downregulated genes mainly included ribosome biogenesis in eukaryotes (5 genes), steroid biosynthesis (2 genes), and protein export (2 genes) (Supplementary figure 1B).

### 3.2. The Expressions of 7 mRNAs Were Identified in DC-CIK Cells from AML Patients

**Figure 3:**
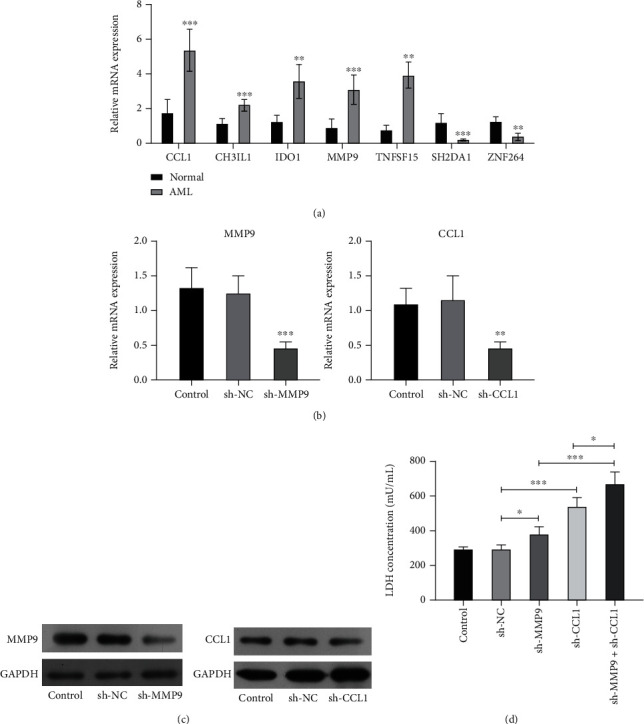
The expressions of 7 mRNAs were identified in DC-CIK cells from AML patients. (a) The levels of the 7 mRNAs with the greatest expression differences were certified through applying the qRT-PCR assay in DC-CIK cells from normal and AML patients. After DC-CIK cells from AML patients were transfected with MMP9 or CCL1 shRNAs, the transfection effects were analyzed via qRT-PCR (b) and western blot assays (c). (d) After MMP9 or CCL1 knockdown, the concentration of LDH was confirmed by applying the LDH kit. ^∗^*P* < 0.05, ^∗∗^*P* < 0.01, and ^∗∗∗^*P* < 0.001.

Moreover, based on the expression changes of genes, we then screened and verified 7 mRNAs (CCL1, CH3IL1, IDO1, MMP9, TNFSF15, SH2DA1, and ZNF264). As displayed in Figure 3(a), we testified that CCL1, CH3IL1, IDO1, MMP9, and TNFSF15 were observably upregulated, and SH2DA1 and ZNF264 were signally downregulated in DC-CIK cells from AML patients relative to that in normal patients. Because the expression of MMP9 and CCL1 in AML patients is higher than that in normal people, in the next study, we explore the functions of MMP9 and CCL1. Afterwards, to further survey the possible functions and mechanisms of MMP9 and CCL1, we adopted MMP9 or CCL1 shRNAs to transfect DC-CIK cells from AML patients. And the results from both qRT-PCR and western blot assays demonstrated that MMP9 and CCL1 expressions were markedly decreased in DC-CIK cells after transfection with respective shRNAs, suggesting that MMP9 and CCL1 were successfully silenced in DC-CIK cells (Figures 3(b) and 3(c)). Simultaneously, we uncovered that MMP9 or CCL1 knockdown memorably elevated the concentration of LDH, and the cotransfection of MMP9 shRNAs and CCL1 shRNAs had a coordinating effect on the elevation of LDH in DC-CIK cells (Figure 3(d)). Studies have reported that LDH is significantly elevated in AML, anemia, malignant lymphoma, and other diseases [36].

### 3.3. MMP9- and CCL1-Silenced DC-CIK Cells Suppressed Proliferation and Induced Apoptosis of Leukemia Cells

**Figure 4:**
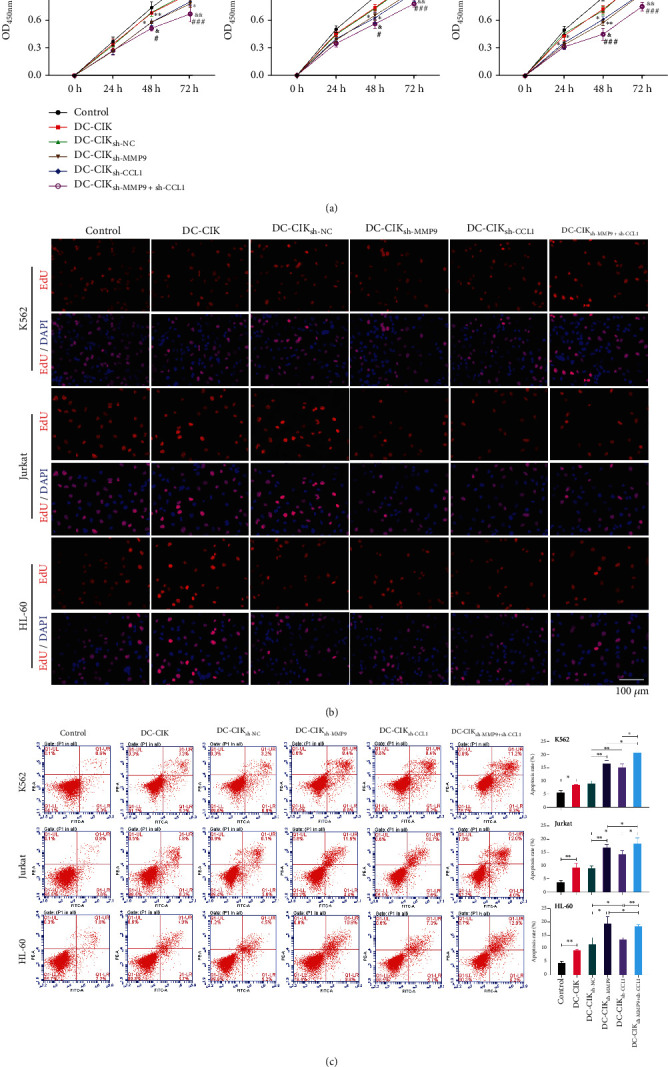
MMP9- and CCL1-silenced DC-CIK cells suppressed proliferation and induced apoptosis of leukemia cells. DC-CIK cells were transfected with MMP9 or/and CCL1 shRNAs and then cocultured with leukemia cells, respectively. Proliferation of the cocultured DC-CIK cells was determined through CCK-8 (a) and EdU staining (b). Magnification, 200x, scale bar = 100 *μ*m. (c) The apoptosis capacity was tested via flow cytometer. ^∗^ means compared to control group, # means compared to DC-CIK shRNA, and & means compared to the DC-CIK+shMMP9 group. Control group means individual tumor cells.

Next, to investigate whether MMP9- and CCL1-silenced DC-CIK cells can significantly affect the proliferation and apoptosis of leukemia cells, MMP9- or/and CCL1-silenced DC-CIK cells were cocultured with 3 leukemia cell lines. The CCK-8 results disclosed that DC-CIK cells effectively repressed the proliferation capacities of leukemia cells, and MMP9 or CCL1 knockdown in DC-CIK cells further prevented the proliferation of leukemia cells relative to that in the DC-CIK_sh-NC_ group. Besides, we also uncovered that the cosilencing DC-CIK cells of MMP9 and CCL1 could further enhance the inhibitory effects of MMP9 or CCL1 knockdown on the proliferation of leukemia cells (Figure 4(a)). Likewise, the EdU staining results also manifested that the proliferation of leukemia cells could be notably suppressed by DC-CIK cells, after MMP9 or/and CCL1 silencing (Figure 4(b)). Furthermore, we confirmed that DC-CIK cells upregulated the apoptotic ratio of leukemia cells. Compared with the DC-CIK_sh-NC_ group, DC-CIK cells after the knockdown of MMP9 or/and CCL1 significantly promoted the apoptosis of leukemia cells (Figure 4(c)).

### 3.4. MMP9- and CCL1-Downregulated DC-CIK Cells Markedly Improved the Immune Function of the Activated T Cells

**Figure 5:**
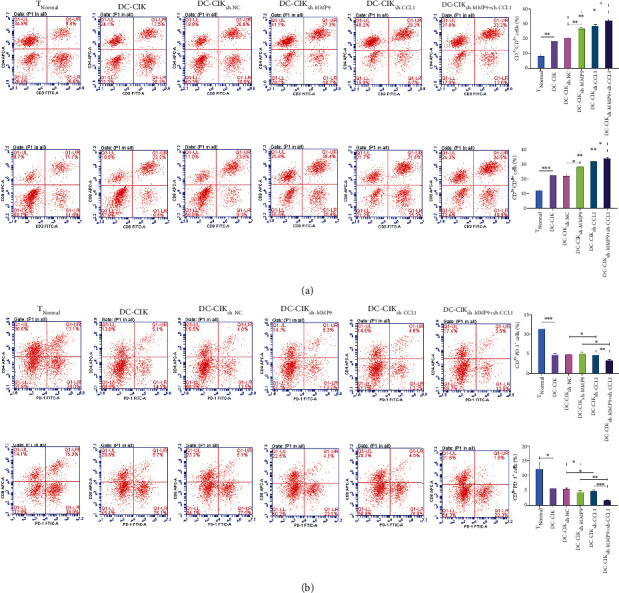
MMP9 and CCL1 downregulated DC-CIK cells markedly improved the immune function of the activated T cells. DC-CIK cells were transfected with MMP9 shRNAs, CCL1 shRNAs, or/and CCL1 plasmid and were cocultured with T cells. (a) The percentages of T-lymphocyte subsets (CD^3*+*^, CD^3*+*^CD^4*+*^, and CD^3*+*^CD^8*+*^) were certified by flow cytometry. (b) The number of the CD4^+^PD-1^+^ and CD8^+^PD-1^+^ cells was determined through flow cytometry. T_Normal_ group means T cells isolated from normal human mononuclear cells.

Further, we also determined the influences of MMP9- and CCL1-silenced DC-CIK cells on the activated T cells. The data of flow cytometry exhibited that DC-CIK cells signally elevated the percentages of T-lymphocyte subsets (CD^3*+*^CD^4*+*^ and CD^3*+*^CD^8*+*^), and this elevation mediated by DC-CIK cells could also be strengthened by MMP9 or CCL1 knockdown; meanwhile, the elevation effect of CD^3*+*^CD^4*+*^ and CD^3*+*^CD^8+^ cells was highest in the cosilencing DC-CIK cells of MMP9 and CCL1 (Figure 5(a)). As well, we also discovered that DC-CIK cells memorably reduced the percentages of CD4^+^PD-1^+^ and CD8^+^PD-1^+^ T cells, the reduction of CD4^+^PD-1^+^ and CD8^+^PD-1^+^ T cells mediated by DC-CIK cells also could be further deepened by MMP9 or CCL1 knockdown in DC-CIK cells, and we also revealed that the reduction of MMP9- and CCL1-silenced DC-CIK cells on the number of CD4^+^PD-1^+^ and CD8^+^PD-1^+^ T cells was most significant (Figure 5(b)).

### 3.5. MMP9- and CCL1-Knockdowned DC-CIK Cells Enhanced T-Cell Activation and Prevented T-Cell Depletion

**Figure 6:**
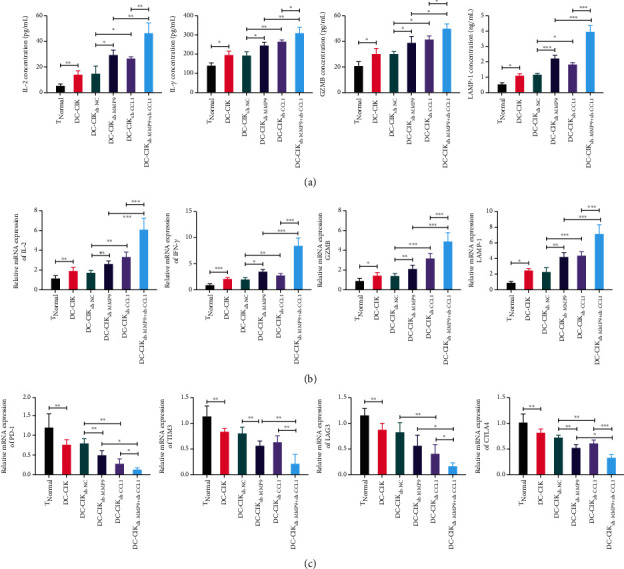
MMP9 and CCL1-knockdowned DC-CIK cells enhanced T-cell activation and prevented T-cell depletion. T cells were cocultured with DC-CIK cells transfected with MMP9 or/and CCL1 shRNAs. (a, b) The levels of T-cell-related cytokines (IL-2, IFN-*γ*, GZMB, and LAMP-1) were monitored through ELISA and qRT-PCR assays. (c) qRT-PCR analysis was conducted to confirm the levels of T-cell depletion indexes (PD-1, TIM3, LAG3, and CTLA4). T_Normal_ group means T cells isolated from normal human mononuclear cells.

Based on the improvement of T-lymphocyte function, we also explored whether MMP9- and CCL1-silenced DC-CIK cells can affect the activation and depletion of T cells. Firstly, the data of ELISA assay displayed that DC-CIK cells signally increased the concentrations of T-cell-related cytokines (IL-2, IFN-*γ*, GZMB, and LAMP-1), which could also be further enhanced by MMP9 or CCL1 knockdown. Besides, when MMP9 and CCL1 were knocked down together, the expressions of IL-2, IFN-*γ*, GZMB, and LAMP-1 showed higher levels than MMP9 or CCL1 knockdown individually in DC-CIK cells (Figure 6(a)). Also, the data of qRT-PCR analysis also exhibited the same expression trends for IL-2, IFN-*γ*, GZMB, and LAMP-1 as that of ELISA assay (Figure 6(b)). Next, we also proved that DC-CIK cells dramatically downregulated T-cell depletion indexes (PD-1, TIM3, LAG3, and CTLA4), which could also be further strengthened by MMP9 or CCL1 knockdown, especially when both MMP9 and CCL1 were knocked down in DC-CIK cells (Figure 6(c)).

### 3.6. Isolated Activated T Cells Suppressed Proliferation and Promote Apoptosis of Leukemia Cells

**Figure 7:**
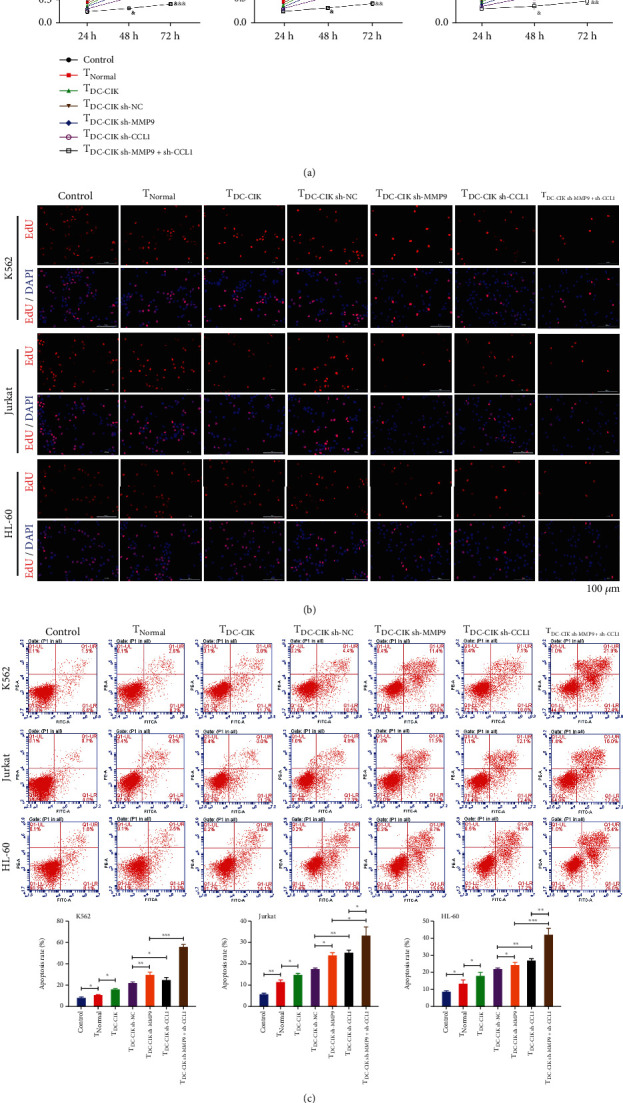
Isolated activated T cells suppressed proliferation and promote apoptosis of leukemia cells. DC-CIK cells were transfected with MMP9 or/and CCL1 shRNA. T cells were isolated from DC-CIK cells, and activated T cells were cocultured with AML cells. Cell proliferation was identified via CCK-8 (a) and EdU staining (b). Magnification, 200x, scale bar = 100 *μ*m. (c) Flow Cytometer was adopted to evaluate the impacts of MMP9 and CCL1 knockdown on the leukemia cells after T-cell activation.

Due to the promotion of MMP9- and CCL1-silenced DC-CIK cells on the T-cell activation, we further verified the effect of coculture of T cells with leukemia cells on leukemia cell proliferation and apoptosis. CCK-8 results disclosed that T cells prominently inhibited the proliferation abilities of leukemia cells, and DC-CIK cell treatment also further reduced the proliferation of leukemia cells after coculture with activated T cells. Simultaneously, we suggested that compared with the DC-CIK cell treatment group, the viability of the cocultured leukemia cells with activated T cells was markedly decreased after treatment with MMP9- and CCL1-silenced DC-CIK cells alone or in combination, especially the MMP9 and CCL1 joint treatment group (Figure 7(a)). Similarly, the results from EdU staining also testified that MMP9- and CCL1-silenced DC-CIK cells displayed the same inhibitory effects on the proliferation of the cocultured leukemia cells with activated T cells (Figure 7(b)). More importantly, our results also uncovered that T cells observably accelerated the apoptosis of leukemia cells. MMP9- and CCL1-silenced DC-CIK cells alone or in combination also further facilitated the apoptosis of the cocultured leukemia cells with activated T cells, especially the combined treatment of MMP9 and CCL1 knockdown in DC-CIK cells (Figure 7(c)).

### 3.7. MMP9- and CCL1-Silenced DC-CIK Cells Notably Ameliorated T-Lymphocyte Function in AML Model Mice

**Figure 8:**
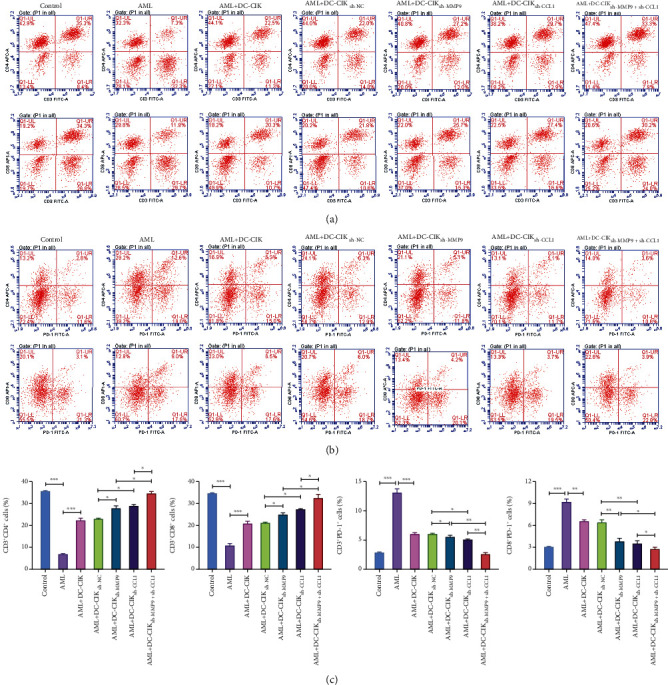
MMP9- and CCL1-silenced DC-CIK cells notably ameliorated T-lymphocyte function in AML model mice. AML model mice were established by injecting HL-60 cells into tail vein and then injected with MMP9- or/and CCL1-silenced DC-CIK cells. (a) Flow cytometry was applied to assess the influences of the modified DC-CIK cells on the T-lymphocyte subsets (CD^3*+*^CD^4*+*^ and CD^3*+*^CD^8*+*^) in AML model mice. (b) Flow cytometry was also utilized to confirm the effects of the modified DC-CIK cells on the number of CD4^+^PD-1^+^ and CD8^+^PD-1^+^ cells in AML model mice. (c) The positive rates of CD^3*+*^CD^4*+*^, CD^3*+*^CD^8*+*^, CD4^+^PD-1^+^, and CD8^+^PD-1^+^ cells were quantitatively analyzed.

Subsequently, we further validated the underlying significance of MMP9- and CCL1-silenced DC-CIK cells on the T-lymphocyte function *in vivo*. We established the AML model mice through injecting with HL-60 cells. We verified that the percentages of CD^3*+*^CD^4*+*^ and CD^3*+*^CD^8+^ cells were memorably reduced in the AML model mice relative to the control mice, while this reduction could be signally attenuated by DC-CIK cells. In addition, we uncovered that MMP9- and CCL1-silenced DC-CIK cells alone or in combination could further enhance the increases of CD^3*+*^CD^4*+*^ and CD^3*+*^CD^8+^ cells mediated by DC-CIK cells (Figures 8(a) and 8(c)). Moreover, our results also exhibited that the percentages of CD4^+^PD-1^+^ and CD8^+^PD-1^+^ cells were observably increased in the AML model mice with respect to the control mice, while this increase could also be dramatically weakened by DC-CIK cells. Besides, we revealed that MMP9- and CCL1-silenced DC-CIK cells alone or in combination also could further strengthen the decreases of CD4^+^PD-1^+^ and CD8^+^PD-1^+^ cells mediated by DC-CIK cells (Figures 8(b) and 8(c)).

### 3.8. MMP9- and CCL1-Downregulated DC-CIK Cells Induced T-Cell Activation and Suppressed T-Cell Depletion in AML Model Mice

**Figure 9:**
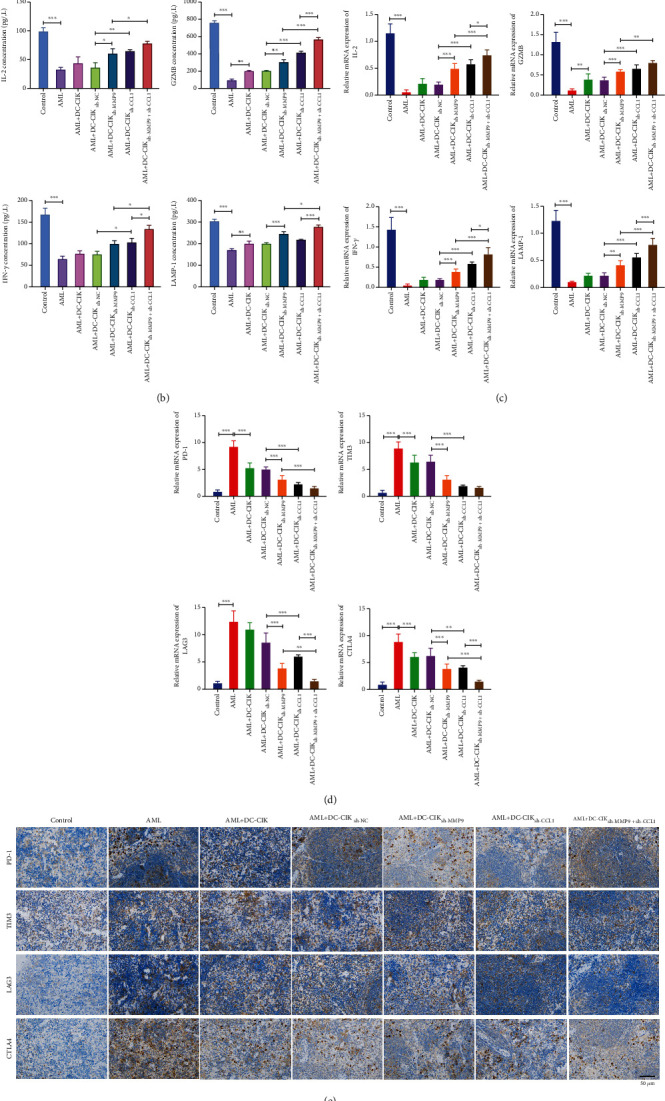
MMP9 and CCL1 downregulated DC-CIK cells induced T-cell activation and suppressed T-cell depletion in AML model mice. MMP9- or/and CCL1-silenced DC-CIK cells were applied to inject the AML model mice through the tail vein. (a) H&E staining displayed the changes in inflammatory infiltration of the spleen in AML model mice. Magnification, 400x, scale bar = 50 *μ*m. (b, c) Both ELISA and qRT-PCR assays were used to examine the changes of T-cell-related cytokines (IL-2, IFN-*γ*, GZMB, and LAMP-1) in AML model mice. The levels of T-cell depletion indexes (PD-1, TIM3, LAG3, and CTLA4) were also analyzed through the application of qRT-PCR (d) and IHC assays (e). Magnification, 400x, scale bar = 50 *μ*m.

Similarly, we also testified the influences of MMP9- and CCL1-silenced DC-CIK cells on the T-cell activation and T-cell depletion in AML model mice. We first analyzed the inflammatory infiltration of spleen tissue of AML model mice by H&E staining. As presented in Figure 9(a), in the control group, the spleen tissues of mice were normal; the morphological structures of red pulp, white pulp, splenic corpuscle, and splenic sinus were intact; and no hyperemia was observed in the splenic sinus. In the AML group, the morphological change of spleen tissue was obvious, the lymphocytes were aggregated in the white medulla with dense coloring, the spleen body was atrophied, and the vascular endothelial cells were swollen. In the AML+DC-CIK group, the degree of spleen tissue injury in mice was slightly improved compared with that in the AML group. DC-CIK cells silenced by MMP9 or CCL1 alone could markedly improve the degree of spleen tissue injury in AML model mice. MMP9- and CCL1-silenced DC-CIK cells also could further alleviate the degree of spleen tissue injury in AML model mice relative to the MMP9-silenced or CCL1-silenced DC-CIK cells. As exhibited in Figure 9(b), the concentrations of IL-2, IFN-*γ*, GZMB, and LAMP-1 were decreased in the AML model mice versus the control mice, while DC-CIK cells could memorably reserve the decreases in the AML model mice; in addition, we disclosed that the increases of IL-2, IFN-*γ*, GZMB, and LAMP-1 levels mediated by DC-CIK cells could be further enhanced by MMP9 or CCL1 knockdown in DC-CIK cells. Above all, MMP9 and CCL1 knockdown had a remarkable coordinating effect on the T-cell-related cytokines. Also, the data of qRT-PCR presented the same trend changes as the ELISA assay (Figure 9(c)). Next, the qRT-PCR and IHC results also proved that the levels of PD-1, TIM3, LAG3, and CTLA4 were signally reduced in the AML model mice compared to the control mice, while this reduction could also be dramatically reserved by DC-CIK cells in the AML model mice; our results also testified that the elevations of PD-1, TIM3, LAG3, and CTLA4 expressions mediated by DC-CIK cells could be further strengthened by MMP9 or/and CCL1 knockdown in DC-CIK cells, in particular the cotransfection of MMP9 and CCL1 shRNAs in DC-CIK cells (Figures 9(d) and 9(e)).

## 4. Discussion

Disease recurrence is still a challenge in the clinical therapy of AML [37]. So far, the intensive treatment after recurrence including chemotherapy or/and immunotherapy remains a vital therapeutic method for AML patients [8]. DC has the functions of uptaking, processing, and presenting antigens and promoting the proliferation abilities of T and B lymphocytes [38, 39]. DC has a significant effect in both innate and acquired immunity, which is particularly important for the treatment of tumors with low endogenous immune response, such as AML [40, 41]. Previous studies have verified that CIK cells can significantly kill a variety of tumor cells such as leukemia [42], multiple myeloma [43], and lymphoma [44, 45]. Researches have disclosed that the coculture of CIK cells with DC can improve the antitumor activity of CIK cells [46, 47]. The study also proved that DC-CIK cells have a more significant antileukemia effect and a stronger killing effect on leukemia cells than CIK cells alone, which brings hope for the treatment of patients with recurrent leukemia [48]. In our study, DC-CIK cells could effectively repress proliferation and accelerate apoptosis of leukemia cells, and DC-CIK cells also could improve the immune function of T cells, induce T-cell activation, and inhibit T-cell depletion in AML cells; meanwhile, we also revealed that DC-CIK cells also could inhibit proliferation and facilitate apoptosis of the cocultured leukemia cells with the activated T cells; above all, *in vivo* experiments also disclosed that DC-CIK cells could ameliorate T-lymphocyte function, induce T-cell activation, and prevent T-cell depletion in AML model mice. Therefore, our experimental results are basically consistent with the previous research results.

Moreover, we adopted RNA sequencing to filtrate the differentially expressed mRNAs (651 upregulated and 151 downregulated genes) in DC-CIK cells from normal and AML patients. Besides, we discovered that the upregulated genes in DC-CIK cells from AML patients, which were mainly located in the intracellular and cytoplasm, were mainly related to the cellular process and response to stimulus; the downregulated genes in DC-CIK cells from AML patients, which were mainly located in the intracellular and organelle, were mainly connected with the metabolic process. Simultaneously, we prompted that the upregulated genes were concentrated in carbon metabolism and glycolysis/gluconeogenesis pathways. The downregulated genes were concentrated in ribosome biogenesis in the eukaryotes pathway. Based on the differential expression and functional annotation of genes, 7 potential genes were filtered and identified in DC-CIK cells from AML patients. We revealed that CCL1, CH3IL1, IDO1, MMP9, and TNFSF15 were upregulated, and SH2DA1 and ZNF264 were downregulated in DC-CIK cells from AML patients. In accordance with the expression difference and literature screening, MMP9 and CCL1 were selected as the research targets in AML.

Matrix metalloproteinases (MMPs) have been verified to be involved in tumor metastasis by the degradation of the extracellular matrix (ECM) [49]. ECM modulation induced by MMPs has the dual effect of suppressing AML cells while improving the retention of healthy hematopoietic stem cells, tilting the competition between healthy and malignant hematopoiesis in favor of the former [50]. Inhibition of MMP expression may complement traditional AML treatment regimens and improve cytopenias associated with the loss of healthy hematopoietic stem and progenitor cells (HSPCs) [50]. MMP9, as one of the crucial proteins, can participate in tumor development processes such as tumor angiogenesis and immune evasion through hydrolysis of adhesion molecules [51]. Study has confirmed that during the pathogenesis of AML, MMP9 can partake in the escape of leukemia cells from the bone marrow via degrading the ECM, leading to the occurrence of extramedullary infiltration [52]. In addition, in this study, we also observed that the expression of MMP9 was significantly correlated with cancer stem cells (Supplementary figure 2). CCL1 is an inflammatory factor associated with the inflammatory response in atopic dermatitis [53]. CCL1 can be secreted by monocytes and expressed on the surface of Th2 cells and Treg cells [54]. Research proved that CCL1 can induce Th2 cellular immune response by activating CCR8, thus inhibiting Th1 cellular immunity [55]. While there is little research on CCL1 in AML. In our study, we further demonstrated that MMP9 and CCL1 knockdown alone or in combination in DC-CIK cells, especially the coknockdown of MMP9 and CCL1 in DC-CIK cells could significantly enhance the roles of DC-CIK cells on the function of AML cells. In addition, we proved that MMP9- and CCL1-silenced DC-CIK cells could further improve T cells, enhance the activation of T cells, and prevent the depletion of T cells in AML cells and AML model mice. Furthermore, we uncovered that MMP9- and CCL1-silenced DC-CIK cells could further prevent the malignant behavior of AML by regulating the activation of T cells.

There is increasing evidence that cellular immunotherapy is a novel treatment for tumors following chemotherapy and hematopoietic stem cell transplantation [56]. CD3+T lymphocytes, CD4+T lymphocytes, and CD8+T levels are often altered in cancer patients. These changes have become a valuable clinical indicator of immunosuppression in treating cancer patients [57]. DC-CIK cell therapy can repeatedly kill and clear occult tumor cells, reducing recurrence and metastasis. The therapy is highly safe and has few side effects on normal tissues [58]. In AML, DC-CIK cells have significantly higher proliferative activity, secreted cytokine levels, and antitumor effects on AML cells than CIK cells [59]. Results of a clinical study showed that the infusion of DC-CIK cells significantly improved the efficacy of chemotherapy in children with AML [60]. Therefore, DC-CIK cell infusion or T-lymphocyte infusion may be a key strategy for antitumor activity. In this study, DC-CIK cells were isolated and cultured from the peripheral blood of normal people and AML patients. RNA sequencing revealed altered levels of multiple mRNAs in DC-CIK cells from AML patients. MMP9 and CCL1 were identified as further study subjects. At the cellular level, the knockdown of MMP9 and CCL1 in DC-CIK cells inhibited the proliferation of AML cells and induced their apoptosis. Additionally, the knockdown of MMP9 and CCL1 in DC-CIK cells significantly enhanced T-cell activation and inhibited T-cell exhaustion. At the animal level, MMP9- and CCL1-silenced DC-CIK cells significantly improved T-lymphocyte function in AML model mice. We systematically verified from different levels that MMP9- and CCL1-silenced DC-CIK cells prevented the malignant development of AML by activating T cells. This is novel and uncommon in the study of AML. This result provides a theoretical and experimental basis for clinical immunotherapy using DC-CIK cells.

## 5. Conclusions

MMP9- and CCL1-silenced DC-CIK cells could significantly prevent the development and progression of AML through activating T cells. Therefore, we testified that MMP9- and CCL1-silenced DC-CIK cells may be widely applied to the treatment of AML in the future.

## Figures and Tables

**Table 1:** Clinical Characteristics of AML Patients.

Characteristics	Median (range)	No. of cases
Sex		
Female		4
Male		8
Total		12
Age		
Median (years)	56.5 (27-76)	
WBC		
Median (×10^9^/L)	15.15 (1.25-221.0)	
Platelets		
Median (×10^9^/L)	71.5 (13.0-247.0)	
AML FAB subtype		
AML with maturation: M2		7
Acute monoblastic or monocytic leukemia: M5		5
Karyotype		
Normal		6
Medium		2
Complex		2
Unknown		2
Gene mutations		
NPM1		4
FLT3-ITD		4
TP53		2
RUNX1		1
CEBPA		1

Abbreviations: AML: acute myeloid leukemia; WBC: white blood cell; FAB subtype: French-American-British subtype; NPM1: nucleophosmin 1; FLT3-ITD: FLT3 internal tandem duplication; TP53: tumor protein p53; RUNX1: runt-related transcription factor 1; CEBPA: CCAAT enhancer binding protein alpha.

**Table 2:** The sequence of all primers in qRT-PCR assay.

ID	Sequence (5′-3′)	Product length (bp)
Human		
GAPDH (forward)	TGTTCGTCATGGGTGTGAAC	154
GAPDH (reversed)	ATGGCATGGACTGTGGTCAT	
CHI3L1 (forward)	GAAGACTCTCTTGTCTGTCGGA	108
CHI3L1 (reversed)	AATGGCGGTACTGACTTGATG	
PD-1 (forward)	CAACGGGCGTGACTTCC	297
PD-1 (reversed)	ATTGTCCCTCGTGCGGC	
MMP9 (forward)	AGACCTGGGCAGATTCCAAAC	94
MMP9 (reversed)	CGGCAAGTCTTCCGAGTAGT	
TNFSF15 (forward)	GCACCTCTTAGAGCAGACGG	212
TNFSF15 (reversed)	CGGAATGTGACCTGGGAGTAAAT	
CCL1 (forward)	CAGCTCCATCTGCTCCAATG	92
CCL1 (reversed)	GCCTCTGAACCCATCCAACT	
IDO1 (forward)	TCTCATTTCGTGATGGAGACTGC	130
IDO1 (reversed)	GTGTCCCGTTCTTGCATTTGC	
ZNF264 (forward)	AGAGTCCGTAGCCATAACTCAT	202
ZNF264 (reversed)	GCAGGAGATGTGTGCTCTTAAT	
SH2D1A (forward)	AGGCGTGTACTGCCTATGTG	183
SH2D1A (reversed)	TGCAGAGGTATTACAATGCCTTG	
TIM3 (forward)	AGACAGTGGGATCTACTGCTG	157
TIM3 (reversed)	CCTGGTGGTAAGCATCCTTGG	
LAG3 (forward)	GCCTCCGACTGGGTCATTTT	131
LAG3 (reversed)	CTTTCCGCTAAGTGGTGATGG	
CTLA4 (forward)	CATGATGGGGAATGAGTTGACC	92
CTLA4 (reversed)	TCAGTCCTTGGATAGTGAGGTTC	
IL-2 (forward)	TCCTGTCTTGCATTGCACTAAG	161
IL-2 (reversed)	CATCCTGGTGAGTTTGGGATTC	
IFN-*γ* (forward)	TCGGTAACTGACTTGAATGTCCA	93
IFN-*γ* (reversed)	TCGCTTCCCTGTTTTAGCTGC	
GZMB (forward)	TACCATTGAGTTGTGCGTGGG	124
GZMB (reversed)	GCCATTGTTTCGTCCATAGGAGA	
LAMP-1 (forward)	TCTCAGTGAACTACGACACCA	151
LAMP-1 (reversed)	AGTGTATGTCCTCTTCCAAAAGC	
Mouse		
*β*-Actin (forward)	CATTGCTGACAGGATGCAGA	139
*β*-Actin (reversed)	CTGCTGGAAGGTGGACAGTGA	
PD-1 (forward)	GGGTATCCCTGTATTGCTGC	147
PD-1 (reversed)	TCCTCATAGGCCACACTAGG	
LAMP-1 (forward)	CAGCACTCTTTGAGGTGAAAAAC	103
LAMP-1 (reversed)	ACGATCTGAGAACCATTCGCA	
GZMB (forward)	TCATGCTGCTAAAGCTGAAGAG	244
GZMB (reversed)	CCCGCACATATCTGATTGGTTT	
IFN-*γ* (forward)	ATGAACGCTACACACTGCATC	182
IFN-*γ* (reversed)	CCATCCTTTTGCCAGTTCCTC	
IL-2 (forward)	TGCGGCATGTTCTGGATTTG	205
IL-2 (reversed)	GGGCTTGTTGAGATGATGCTT	
CTLA4 (forward)	CCATACAGGTGACCCAACCT	283
CTLA4 (reversed)	ACCTTGCAGAGGTACAGTCC	
LAG3 (forward)	CTCAATGCCACTGTCACGTT	94
LAG3 (reversed)	GGGTTACCTCACACAACAGC	
TIM3 (forward)	AGGGCGATCTCAACAAAGGA	163
TIM3 (reversed)	GTCTGAGCTGGAGTGACCTT	

## Data Availability

All data generated or analyzed during this study are included in this published article.

## Supplementary Materials

Supplementary 1 Supplementary figure 1: (A) the top 10 BP, CC, and MF of the upregulated (up) and downregulated (down) mRNAs were exhibited through the GO analysis. (B) The top 10 signaling pathways enriched by the upregulated (up) and downregulated (down) mRNAs were shown using the KEGG analysis.

Supplementary 2 Supplementary figure 2: correlation between MMP9 gene expression and cancer stem cells.
