# Supplementary material for: MMP9-Associated Tumor Stem Cells, CCL1-Silenced Dendritic Cells, and Cytokine-Induced Killer Cells Have a Remarkable Therapeutic Efficacy for Acute Myeloid Leukemia by Activating T Cells
Source: Stem Cells Int. 2023 May 9;2023:2490943. doi: 10.1155/2023/2490943 (PMC10188259; doi:10.1155/2023/2490943)
Supplement: Supplementary 1 — Supplementary figure 1: (A) the top 10 BP, CC, and MF of the upregulated (up) and downregulated (down) mRNAs were exhibited through the GO analysis. (B) The top 10 signaling pathways enriched by the upregulated (up) and downregulated (down) mRNAs were shown using the KEGG analysis. [file 2490943.f1.pdf]

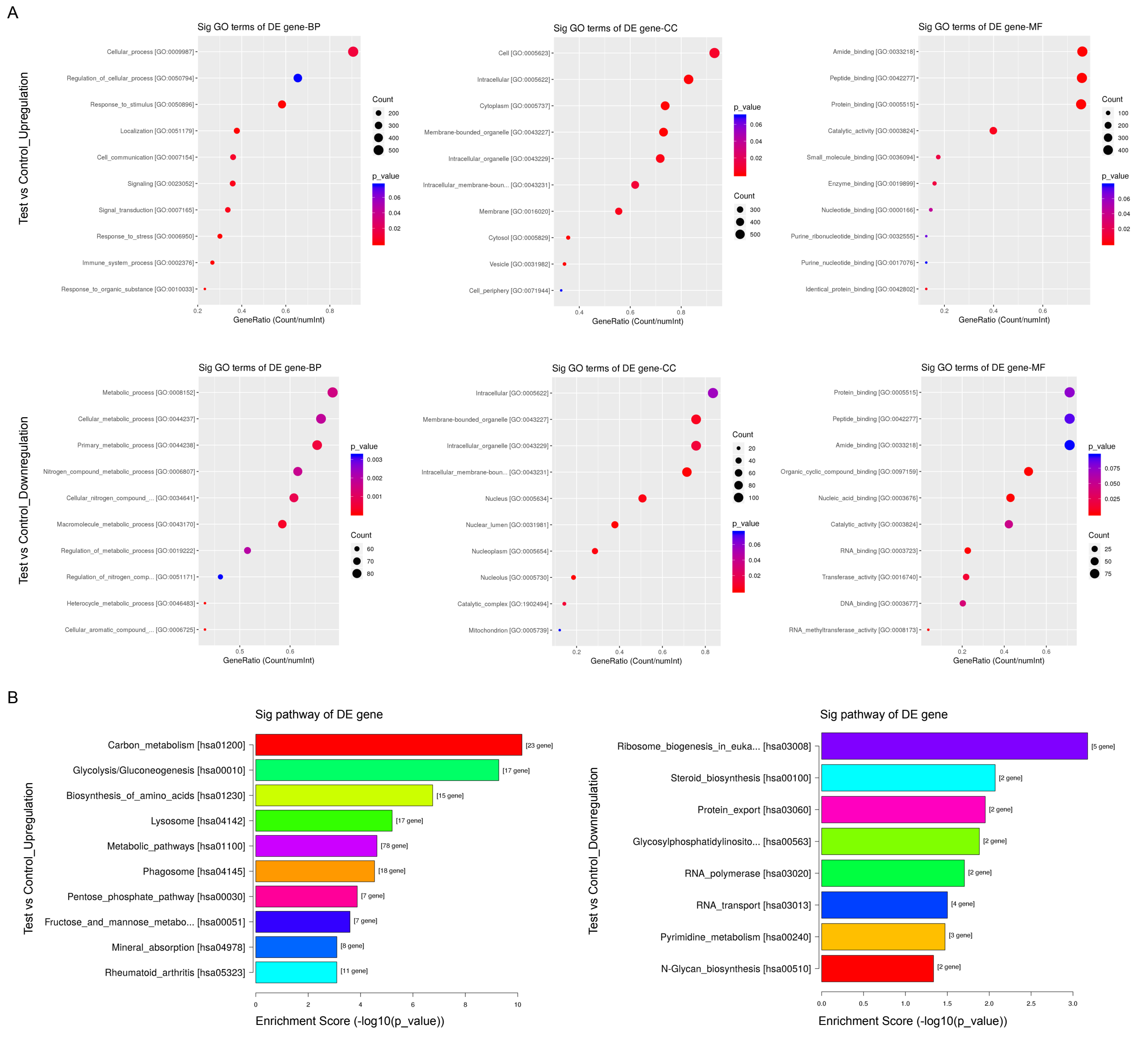

A
Test vs Control_Upregulation
Sig GO terms of DE gene-BP
Cellular_process [GO:0009987]
Regulation_of_cellular_process [GO:0050794]
Response_to_stimulus [GO:0050896]
Localization [GO:0051179]
Cell_communication [GO:0007154]
Signaling [GO:0023052]
Signal_transduction [GO:0007165]
Response_to_stress [GO:0006950]
Immune_system_process [GO:0002376]
Response_to_organic_substance [GO:0010033]
GeneRatio (Count/numInt)
Sig GO terms of DE gene-CC
Cell [GO:0005623]
Intracellular [GO:0005622]
Cytoplasm [GO:0005737]
Membrane-bounded_organelle [GO:0043227]
Intracellular_organelle [GO:0043229]
Intracellular_membrane-boun... [GO:0043231]
Membrane [GO:0016020]
Cytosol [GO:0005829]
Vesicle [GO:0031982]
Cell_periphery [GO:0071944]
Sig GO terms of DE gene-MF
Amide_binding [GO:0033218]
Peptide_binding [GO:0042277]
Protein_binding [GO:0005515]
Catalytic_activity [GO:0003824]
Small_molecule_binding [GO:0036094]
Enzyme_binding [GO:0019899]
Nucleotide_binding [GO:0000166]
Purine_ribonucleotide_binding [GO:0032555]
Purine_nucleotide_binding [GO:0017076]
Identical_protein_binding [GO:0042802]
Count
p_value
Test vs Control_Downregulation
Sig GO terms of DE gene-BP
Metabolic_process [GO:0008152]
Cellular_metabolic_process [GO:0044237]
Primary_metabolic_process [GO:0044238]
Nitrogen_compound_metabolic_process [GO:0006807]
Cellular_nitrogen_compound_... [GO:0034641]
Macromolecule_metabolic_process [GO:0043170]
Regulation_of_metabolic_process [GO:0019222]
Regulation_of_nitrogen_comp... [GO:0051171]
Heterocycle_metabolic_process [GO:0046483]
Cellular_aromatic_compound_... [GO:0006725]
Sig GO terms of DE gene-CC
Intracellular [GO:0005622]
Membrane-bounded_organelle [GO:0043227]
Intracellular_organelle [GO:0043229]
Intracellular_membrane-boun... [GO:0043231]
Nucleus [GO:0005634]
Nuclear_lumen [GO:0031981]
Nucleoplasm [GO:0005654]
Nucleolus [GO:0005730]
Catalytic_complex [GO:1902494]
Mitochondrion [GO:0005739]
Sig GO terms of DE gene-MF
Protein_binding [GO:0005515]
Peptide_binding [GO:0042277]
Amide_binding [GO:0033218]
Organic_cyclic_compound_binding [GO:0097159]
Nucleic_acid_binding [GO:0003676]
Catalytic_activity [GO:0003824]
RNA_binding [GO:0003723]
Transferase_activity [GO:0016740]
DNA_binding [GO:0003677]
RNA_methyltransferase_activity [GO:0008173]
B
Sig pathway of DE gene
Test vs Control_Upregulation
Carbon_metabolism [hsa01200] [23 gene]
Glycolysis/Gluconeogenesis [hsa00010] [17 gene]
Biosynthesis_of_amino_acids [hsa01230] [15 gene]
Lysosome [hsa04142] [17 gene]
Metabolic_pathways [hsa01100] [78 gene]
Phagosome [hsa04145] [18 gene]
Pentose_phosphate_pathway [hsa00030] [7 gene]
Fructose_and_mannose_metabo... [hsa00051] [7 gene]
Mineral_absorption [hsa04978] [8 gene]
Rheumatoid_arthritis [hsa05323] [11 gene]
Enrichment Score (-log10(p_value))
Test vs Control_Downregulation
Ribosome_biogenesis_in_euka... [hsa03008] [5 gene]
Steroid_biosynthesis [hsa00100] [2 gene]
Protein_export [hsa03060] [2 gene]
Glycosylphosphatidylinosito... [hsa00563] [2 gene]
RNA_polymerase [hsa03020] [2 gene]
RNA_transport [hsa03013] [4 gene]
Pyrimidine_metabolism [hsa00240] [3 gene]
N-Glycan_biosynthesis [hsa00510] [2 gene]
Enrichment Score (-log10(p_value))
