# Supplementary figures and images for: MMP9-Associated Tumor Stem Cells, CCL1-Silenced Dendritic Cells, and Cytokine-Induced Killer Cells Have a Remarkable Therapeutic Efficacy for Acute Myeloid Leukemia by Activating T Cells
Source: Stem Cells Int. 2023 May 9;2023:2490943. doi: 10.1155/2023/2490943 (PMC10188259; doi:10.1155/2023/2490943)

**MMP9 Expression**

$p = 0.018$ ,  $\hat{r}_{\text{Pearson}} = 0.22$ ,  $\text{CI}_{95\%} [0.04, 0.38]$ ,  $n_{\text{pairs}} = 120$

0.2

0.3

0.4

0.5

0.6

**Tumor stem cells score**

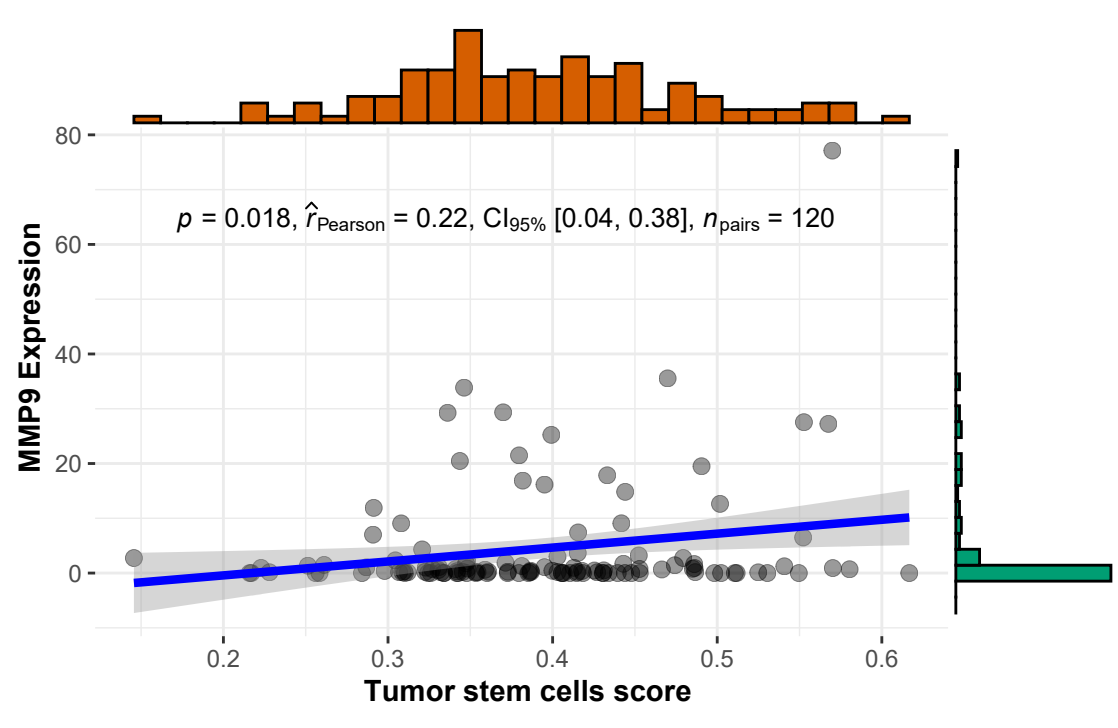

Supplement: Supplementary 2 — Supplementary figure 2: correlation between MMP9 gene expression and cancer stem cells. [file 2490943.f2.pdf]
